# Investigation of Changes in Saliva in Radiotherapy-Induced Head Neck Cancer Patients

**DOI:** 10.3390/ijerph18041629

**Published:** 2021-02-09

**Authors:** Christina Winter, Roman Keimel, Markus Gugatschka, Dagmar Kolb, Gerd Leitinger, Eva Roblegg

**Affiliations:** 1 Institute of Pharmaceutical Sciences, Pharmaceutical Technology and Biopharmacy, University of Graz, Universitätsplatz 1, 8010 Graz, Austria; christina.winter@uni-graz.at (C.W.); roman.keimel@uni-graz.at (R.K.); 2 Research Center Pharmaceutical Engineering GmbH, Inffeldgasse 13, 8010 Graz, Austria; 3 Division of Phoniatrics, Medical University of Graz, Auenbruggerplatz 26, 8036 Graz, Austria; markus.gugatschka@medunigraz.at; 4 BioTechMed-Graz, 8010 Graz, Austria; 5 Core Facility Ultrastructure Analysis, Center for Medical Research, Gottfried Schatz Research Center, Medical University of Graz, Neue Stiftingtalstrasse 6/II, 8010 Graz, Austria; dagmar.kolb@medunigraz.at; 6 Division of Cell Biology, Histology and Embryology, Gottfried Schatz Research Center, Medical University of Graz, Neue Stiftingtalstrasse 6/II, 8010 Graz, Austria; gerd.leitinger@medunigraz.at

**Keywords:** UWS, radiation therapy, xerostomia, oral pathology, oral rehabilitation adhesion, hyaluronic acid

## Abstract

The intact function of the salivary glands is of utmost importance for oral health. During radiotherapy in patients with head and neck tumors, the salivary glands can be damaged, causing the composition of saliva to change. This leads to xerostomia, which is a primary contributor to oral mucositis. Medications used for protective or palliative treatment often show poor efficacy as radiation-induced changes in the physico-chemical properties of saliva are not well understood. To improve treatment options, this study aimed to carefully examine unstimulated whole saliva of patients receiving radiation therapy and compare it with healthy unstimulated whole saliva. To this end, the pH, osmolality, electrical conductivity, buffer capacity, the whole protein and mucin concentrations, and the viscoelastic and adhesive properties were investigated. Moreover, hyaluronic acid was examined as a potential candidate for a saliva replacement fluid. The results showed that the pH of radiation-induced saliva shifted from neutral to acidic, the osmolality increased and the viscoelastic properties changed due to a disruption of the mucin network and a change in water secretion from the salivary glands. By adopting an aqueous 0.25% hyaluronic acid formulation regarding the lost properties, similar adhesion characteristics as in healthy, unstimulated saliva could be achieved.

## 1. Introduction

Saliva is of utmost importance for oral health, as it performs a variety of vital functions [1]. It consists of water, enzymes, electrolytes and proteins that work together to assist in swallowing, digestion, protection, moisturization and other tasks [2,3]. Saliva is produced by the major salivary glands (submandibular, sublingual and parotid) and the minor glands, which are innervated by the autonomic nervous system [4]. External factors, such as radiotherapy treatment of head and neck cancer patients, can have a negative effect on salivary glands and saliva production [5].

Head and neck cancer is the sixth most common cancer worldwide. Approximately 630,000 patients are diagnosed each year and the annual death rate is 350,000 [6]. After diagnosis, treatment depends on the tumor, more precisely on the type, stage and location of the tumor [7]. Surgical resection is usually performed, followed by radiotherapy. Depending on the location of the tumor, radiation can also damage healthy tissues such as the salivary glands, leading to secondary side-effects, namely xerostomia (dry mouth), dysphagia, malnutrition, loss of taste, oral mucositis, and oral infections [7,8,9]. Thereby, xerostomia is the most common side-effect and a contributory cause of oral mucositis and dysphagia. Due to the high sensitivity of the salivary glands to radiation, the salivary flow rate is decreased by 50–60% within the first week of treatment [7,10,11]. This also causes a change in the composition of saliva. It becomes more viscous, the electrolyte levels change, the buffer capacity is reduced, and the pH shifts from neutral to acidic [2,12,13,14,15]. Moreover, it is assumed that crucial components like the mucoglycoproteins or mucins, which determine the viscoelastic behavior of saliva and aid lubrication and adhesion to the underlying epithelium, show changes [16,17,18,19]. The most frequently occurring mucins in human saliva are MUC5B and MUC7, which are produced in the submandibular and sublingual glands [20]. Both mucins are highly C-, N-, and O-glycosylated. MUC5B has a core peptide [21] consisting of a repetitive sequence of proline, alanine, thymine, serine and lysine, and 40 to 80% of O-linked oligosaccharides side chains, such as N-acetylglucosamine, galactose, fucose, and N-acetyl galactosamine [22,23]. Due to calcium-mediated cross-linking and interactions between carbohydrates and hydrophobic groups, MUC5B forms a network responsible for the viscoelastic properties of saliva [24]. Together with secretory immunoglobulin A, cystatin, MUC7 and MUC1, which acts as a connection between the epithelium and MUC5B, it forms the mucosal pellicle, a thin layer of residual saliva that protectively covers the oral soft tissue.

Studies on the regulation of mucin excretion during chemo- and radiotherapy report that mucin concentration increases with increasing treatment cycles [21]. Furthermore, it is assumed that after radiotherapy, MUC5B levels vary depending on the xerostomia level [25]. In non-radiation-induced patients who suffered from dry mouth (e.g., Sjögren syndrome), it was found that MUC5B was still present on the inner lining despite a zero flow rate [26,27]. However, although the mucin concentration did not change, patients showed less hydration and reduced saliva spinnability caused by the degradation of the charged glycans [27]. Moreover, Alliende et al. found that also sulfation is decreased [28]. These findings suggest that the protective salivary mucin barrier is changing.

Currently, treatment management for radiation-induced xerostomia is limited and can be divided into protective and palliative therapies. For example, a medication that protects the salivary glands is amifostine, an oxygen scavenger [29,30]. The disadvantage of amifostine is that it is administered intravenously and leads to severe side-effects including excessive sweating, nausea, bronchoconstriction, hypotension, and bradycardia; hence, patient acceptance is low [31,32]. Growth factors also act as cytoprotectives. Palifermin (Kepivance), a keratinocyte growth factor-1 approved by the Food and Drug Administration in 2015, reduces the risk of developing severe mucositis and shortens the duration of severe mucositis caused in patients receiving high doses of chemo- and radiotherapy followed by stem cell rescue. Basic fibroblast growth factors are under investigation as well as endothelial and epithelial growth factors (EGF) [7,33]. EGF is an amino acid polypeptide found in various biological fluids, including human saliva. It maintains the epithelial barrier by stimulating cell proliferation and has a cytoprotective effect on tissue damage [22,34,35,36]. Epstein et al. suggest that the amount of EGF in the saliva of head neck cancer patients is one of the key factors in predicting the severity of oral mucositis, as it decreases during radiation treatment [34]. Palliative treatments include muscarinic agonists, such as pilocarpine or cevimeline that cause various side-effects [29,37,38]. They are usually applied orally, which often becomes impossible for patients in the advanced oral mucositis stage due to swallowing problems. Another therapeutic approach includes topical palliative agents such as saliva substitutes, mouth rinses, or gels [30,39,40,41]. These formulations consist mainly of polymers such as polyethylene glycol, methylcellulose, chitosan or xylitol [42,43,44,45]. Another well-described polymer candidate that supports cell proliferation, anti-inflammatory processes and consequently wound healing is hyaluronic acid (HA) [30,46]. It is a natural polysaccharide consisting of glucuronic acid and N-acetylglucosamine units. HA is an endogenous substance produced in the serous glands of the submucosa [47]. It is an essential component of the extracellular matrix of the connective tissue and also exhibits antioxidant, antibacterial, antifungal and mucoadhesive effects [48]. Puccio et al. confirmed the adhesive and tissue-repairing properties of aqueous HA solutions in-vitro using a human fibroblast cell line and porcine excised vaginal mucosa [49]. The healing effect of HA in the course of oral mucositis is not entirely understood and two mechanisms of action are discussed. The first assumption is that an adherent layer is formed between the oral environment and the mucosa, leading to reduced pain and healing of the superficial tissue [50]. The second and more likely one is the involvement of biomolecular and physiological changes in keratinocytes and fibroblasts induced by HA [51]. In addition, interactions between HA and toll-like receptors (TLRs) I and II have been reported that prevent penetration of bacteria and viruses into ulcerated tissue [47].

In order to advance the development of therapeutics for the treatment of radiation-induced xerostomia and consequently mucositis, the aim of this work was to carefully examine unstimulated whole saliva of radiation-induced head and neck cancer patients (UWS_RT_) and compare it to healthy unstimulated whole saliva (UWS). To this end, pH, osmolality, electrical conductivity and buffer capacity were studied and the whole protein and mucin concentrations were determined. Viscoelastic and adhesive properties were analyzed, and the salivary mucin network structure was visualized using a cryo-scanning electron microscopy (SEM) technique. For the applicability of HA as a saliva replacement candidate, the pH, osmolality, viscoelastic properties, adhesion and the micro-network were adjusted according to the obtained parameters.

## 2. Materials and Methods

### 2.1. Saliva Collection

UWS was obtained from healthy male and female volunteers (age 25 to 50 years, *n* = 8). Samples were collected according to a standard protocol between 8 a.m. and 9 a.m. [52]. At least one hour before collection, no food, drink or oral hygiene measures were taken. Participants were asked to rinse the mouth with water for one minute and to rest for five minutes before saliva was collected by drooling in sterile tubes without using stimulation to increase salivary flow. The samples were immediately stored on ice and transferred to the refrigerator. Finally, 30-min centrifugation at 2000 rpm at 4 °C was performed to remove residual cells [44,52]. UWS_RT_ was collected from male and female patients (age 21 to 79 years, *n* = 40) under radiation therapy in the head and neck area. Procedures for collection, storage and purification of UWS_RT_ were identical to those described above. No stimulation was performed before or during collection. Unless otherwise indicated, UWS or UWS_RT_ samples from three subjects were pooled for each analytical method.

The study was approved by the Ethics Committee at the Medical University Graz (EK 29-624 ex 16/17). Study procedures were followed in accordance with the Helsinki Declaration. Informed consent was obtained from each subject prior to entry into this study.

### 2.2. Preparation of the HA Solution

HA (hyaluronic acid sodium salt from Streptococcus equi, Mw 50–70 kDa, Sigma Aldrich, Germany) was dispersed in MiliQ-water to reach a concentration of 0.25% (*w*/*w*) and the mixture was stirred at 250 rpm at room temperature until a clear solution was obtained.

### 2.3. Physico-Chemical Characterization

#### 2.3.1. pH-Meter, Freezing Point Depression, Conductivity and Buffer Capacity

UWS and UWS_RT_ were characterized regarding pH using a pH-meter (Lab 860, Schott Instruments, USA, calibrated between pH 4 and 9) at 25 °C. Osmolality was determined via freezing point depression according to the manual (Osmomat O30-D Gonotec, Berlin, Germany), and electrical conductivity measurements were carried out with a conductivity-measuring instrument (WA-100 A-TC, Hirschau, Germany) at 25 °C. The buffer capacity of UWS and UWS_RT_ was measured via acid titration [53,54,55]. To this end, 1 mL UWS or UWS_RT_ were placed in a closed glass vial (20 mL volume) and 0.01 M HCl was added dropwise with a burette through a small opening adapted to the burette to avoid uncontrolled CO_2_ loss. The pH changes were monitored over a range from initial pH (pHi) to pH 4. All samples were continuously stirred (150 rpm) at room temperature during the process.

The buffer capacity of all samples was calculated using the Van Slyke formula:

(1) β=ΔCa/(ΔpH)

where β is the buffer capacity (mol/ĺpH, defined as slyke), ΔCa (mol/L) as the amount of acid added to each pH-step and ΔpH as the change in pH induced by the acid addition [44,53,55].

#### 2.3.2. Protein Concentrations Using BCA Protein Assays

UWS and UWS_RT_ without prior dialysis were used to determine the total protein concentration. For the determination of the mucin concentration in UWS and UWS_RT_ respectively, dialysis was performed using a cellulose acetate membrane (MW cut-off of 12–14 kDa, Carl Roth, Germany) in 2000 mL 50mM NaCl for 12 h to separate the high molecular weight proteins [56].

Salivary whole protein and mucin concentration were determined using a standard Pierce BCA Protein Assay Kit (Thermo Scientific™ Pierce™, Waltham, MA, USA). This assay uses the state-of-the-art method of reducing Cu^2+^ to Cu^+1^ when in contact with proteins in an alkaline medium. The amount of Cu^+1^ can then be assessed via color reaction upon bicinchoninic acid addition at 562 nm. Diluted Albumin served as a standard reagent and Milli-Q^®^-water as blank. The BCA assay was carried out according to the standard protocol. Briefly, 25 µL of both UWS and UWS_RT_ (each *n* = 6) were mixed with 200 µL working reagent in a 96-well-plate. The plate was shaken for 30 s on a plate shaker and incubated for 30 min at 37 °C under light exclusion. Absorbance was measured using a UV-/VIS plate reader (Fluostar Galaxy, BMG Labtech, Ortenberg, Germany) at 562 nm.

#### 2.3.3. Viscoelasticity and Adhesion Test

The viscoelastic behavior (storage modulus G′, loss modulus G″ and complex viscosity η*) was investigated with a Physica MCR 301 rheometer (Anton Paar, Graz, Austria) using a CP-50–1 measurement system (cone-plate geometry) at 25 °C. For oscillation measurements, shear rates between 0.1 to 10 rad/s were applied to simulate naturally occurring shear rates in the oral cavity during swallowing. To prevent liquid evaporation and adsorption of protein molecules at the periphery of the measurement system, a build-in evaporation hood was used. Further, 10 µL 0.1% sodium dodecyl sulfate (SDS, Sigma-Aldrich, Darmstadt, Germany) were applied around the measuring gap to avoid adsorption of protein molecules at the geometry’s periphery [52]. The loss factor tanδ was calculated as the ratio of G″/G′.

Adhesion was investigated via the tack-test at 25 °C using the same rheometer with a PP-25 measurement system (plate-plate geometry). In a typical tack test for gel compounds, a rigid probe is brought into contact with the sample and a constant force is applied [44,49,57,58,59]. After a defined period of time, the probe and the sample are separated at a constant rate while measuring the normal force (F_N_) at a specific distance and duration required for the separation. Furthermore, the maximum force (F_max_) for detachment as well as the overall shape of the force curve, which is determined by the viscoelastic and molecular properties of polymeric components, are used for interpretation of the adhesive behavior [49,59,60]. To adjust our experimental set-up to the constant shear stress in the oral cavity during swallowing, oscillation was applied prior to separation. The measurement gap was adjusted to 0.2 mm to ensure contact between UWS or HA, and shear rates between 0.1 to 10 rad/s were applied before the stainless steel probe was separated at a speed of 500 µm/s from the sample. During the separation time, 500 data points were used to evaluate F_N_ as well as F_max_, which is usually converted and expressed to a positive value [49,61].

#### 2.3.4. Cryo-SEM

The mucin structure of UWS and UWS_RT_ formed by mucins was visualized via Cryo-SEM technique (Quorum PP3010T, Quorum Technologies, Laughton, East Sussex, UK). Prior to visualization, the samples were frozen under slush liquid nitrogen and transferred with a vacuum transfer device into the preparation chamber before subsequent processing and observation. The preparation chamber was connected to a GEMINI Sigma 500 (ZEISS Company, Oberkochen, Germany) SEM, which included a nitrogen gas cold stage. The samples were fractured, sublimated, and sputter-coated with palladium in the chamber. The fractured material was transferred into the SEM specimen chamber before image recording. Images were made with a backscattered electron detector at magnification between 10 kx and 20 kx. To obtain an estimate of the pore size distribution, we used a state-of-the-art mathematical model to calculate the Ferret diameters of irregularly shaped structures to allow comparability with previous studies [44,52,62,63,64]. To this end, the two-dimensional images were converted into binary images using the threshold function of ImageJ-Fiji-software package. From the inverted binary files, the Feret diameters of at least 100 pores of UWS, UWS_RT_ and HA 0.25% were calculated [52,63,65]. To determine the pore size, each pore was assumed to be a spherical particle and calculated according to Equation (2).

(2) $$V\;=\;\frac{4}{3}\;\times \pi \;\times \;(\frac{d}{2}{)}^{3}$$

The pore–size distribution was expressed as volume percentage to consider large and small pores accordingly.

### 2.4. Statistical Analysis

Depending on the respective method, triple or six-fold determinations were carried out. The results are presented as mean values ± standard deviation. To evaluate statistical significance between the characteristics of UWS and UWS_RT_ unpaired Student’s *t*-test was used. Differences were evaluated significant at a level of *p* < 0.05, (*), *p* < 0.01 (**) and *p* < 0.001 (***).

## 3. Results

### 3.1. Study Design

The radiotherapy fractions received by the patients ranged from 8 to 35. For pore size determination two fractions, i.e., UWS_RT8-16_ and UWS_RT25-28_ were used. UWS_RT8-16_ was sampled between the 8th and 16th fraction of radiation therapy and UWS_RT25-28_ between the 25th and 28th fraction. The inclusion criteria of the study included radiotherapy patients, who experienced a feeling of dryness or pain in the oral cavity and showed a rating scale of 1 to 3 for oral mucositis. Patients suffering from severe ulcerations with bleeding or fungal infections were not recruited into the study. Eight patients were excluded because the sample volume obtained was too small for further analysis. Consequently, the total number of patients tested was *n* = 32.

### 3.2. Physico-Chemical Characteristics of UWS, UWS_RT_ and HA

All data are listed in Table 1. The results of UWS_RT_ showed that already in the first two weeks of radiotherapy changes in the physico-chemical properties occurred. The pH decreased from 6.76 ± 0.19 to 6.01 ± 0.68 and osmolality significantly increased from 0.050 ± 0.013 to 0.165 ± 0.056 osmol/kg. There was no significant change found in the electrical conductivity, the value of UWS_RT_ was slightly higher than UWS. The total buffer capacity (pHi to pH 4) of UWSRT decreased only slightly compared to UWS from 5.34 ± 1.7 mmol H^+^/L (UWS) to 4.44 mmol± 0.56 mmol H^+^/L. For the 0.25% aqueous HA solution the results were comparable to UWS. The pH was 7.89 ± 0.14 and the osmolality remained in the low hypotonic range (i.e., 0.018 * ± 0.0008).

#### 3.2.1. Protein and Mucin Concentrations in UWS Versus UWS_RT_

**Figure 1:**
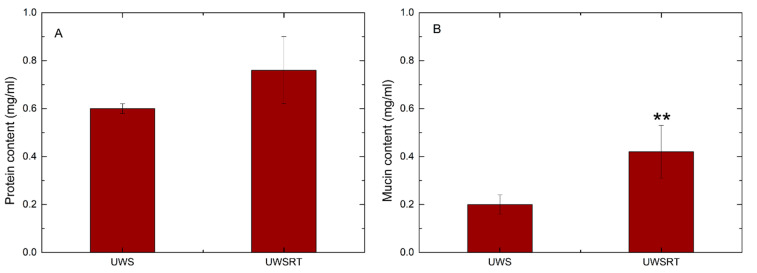
Comparison of the whole protein concentration (**A**) and mucin concentration (**B**) of UWS (healthy volunteers) and UWS_RT_ (patients receiving radiotherapy). Differences were evaluated significant at a level of *p* < 0.01 (**).

The whole protein concentration was higher in UWS_RT_ than in UWS (0.75 ± 0.24 mg/mL vs. 0.60 ± 0.02 mg/mL), although the difference was not significant. Regarding the mucin concentration, there was a significant increase for UWS_RT_ (0.44 mg/mL) compared to UWS (0.19 mg/mL) from healthy volunteers (Figure 1).

#### 3.2.2. Viscoelasticity and Adhesion Test

**Figure 2:**
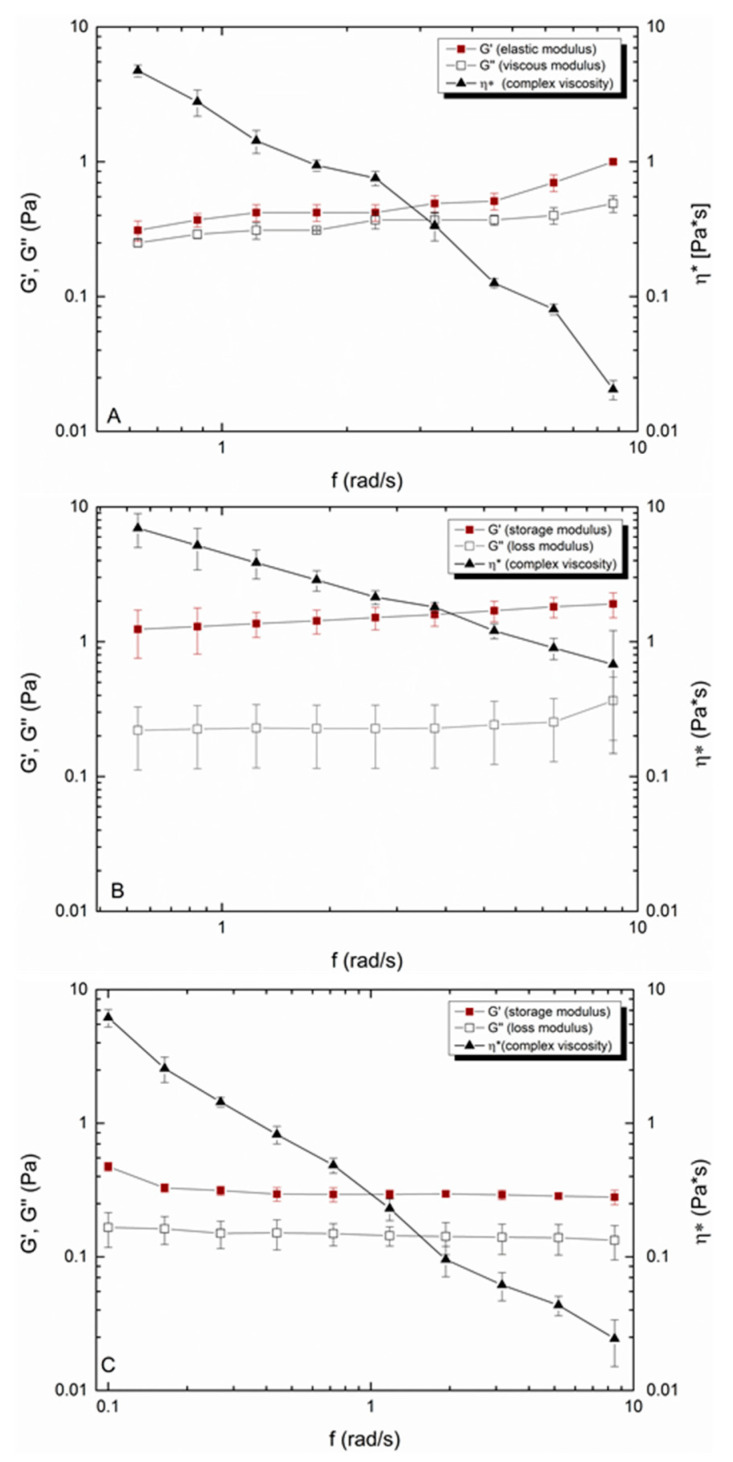
Comparison of the rheological properties of UWS from healthy volunteers (**A**), UWS_RT_ from patients receiving radiotherapy (**B**) and the HA 0.25% (*w*/*w*) (**C**).

For UWS, it was found that the elastic modulus G′ dominated the viscous modulus G″ over the investigated shear rates (Figure 2A). The value for the lowest shear rate of G′ was 0.25 ± 0.03 Pa and for the highest 0.49 ± 0.07 Pa. The loss factor, which is the ratio of the viscoelastic moduli, was constant over the measured range and was between 0.81 to 0.89. This indicates that UWS is a slightly crosslinked viscoelastic fluid (tanδ = 1). For UWS_RT_ the viscoelastic moduli were significantly higher (Figure 2B). The elastic modulus G′ increased by a factor of twelve from 0.31 ± 0.03 Pa to 3.79 *** ± 0.42 Pa at the lowest shear rate and from 0.89 ± 0.19 Pa to 5.03 ***± 0.78 Pa at the highest shear rate. The viscous modulus G″ also increased to 0.99 *** ± 0.12 Pa and 1.52 ***± 0.21 Pa respectively at lower and higher shear rates. Since G′ increased more strongly than G″, the loss factor tanδ changed accordingly, suggesting that UWS_RT_ is a viscoelastic solid with a reduced fluid portion left (0.55 ± 0.12 to 0.65 ± 0.09 over the applied shear range). Both UWS and UWS_RT_ showed a shear-thinning behavior and the viscosity of UWS_RT_ was increased over the whole measurement range.

Similar viscoelastic behavior was obtained for HA 0.25% (*w*/*w*) after stepwise dilution of a 1% HA solution (data not shown). The elastic modulus G′ was larger than G″ over the applied shear range with calculated tanδ values between 0.69 at low and 0.79 at higher shear rates (Figure 2C). The shear-thinning fluid showed a similar initial viscosity (7.19 ± 0.12 Pa*s) than UWS (5.23 ± 0.49 Pa*s), and at higher shear rates, the shear-thinning effect was almost identical (Figure 2).

**Figure 3:**
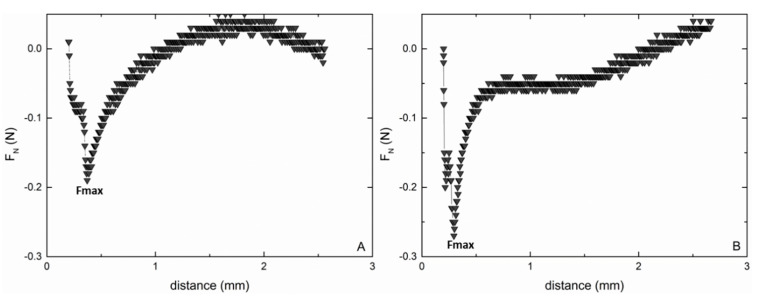
Representative force distance curves for UWS (**A**) and HA 0.25% (*w*/*w*) (**B**) gained from tack tests.

The force curves gained from the tack test showed the adhesive and cohesive behavior of both UWS and HA (Figure 3A,B). During the tack test, the mucin and polymeric chains were stretched and therefore put under increasing stress, followed by fibril fracture and cohesive debonding [60]. F_max_ of UWS before the disruption point was 0.21 ± 0.02 N while HA 0.25% (*w*/*w*) displayed a sharper defined detachment curve with a F_max_ of 0.28 ± 0.08 N. The full disruption process (zero force) for HA 0.25% (*w*/*w*) was completed after 2.6 ± 0.02 mm, while for UWS a steeper course of the curve was observed (zero force at 1.21 ± 0.11 mm).

#### 3.2.3. Cryo-SEM

**Figure 4:**
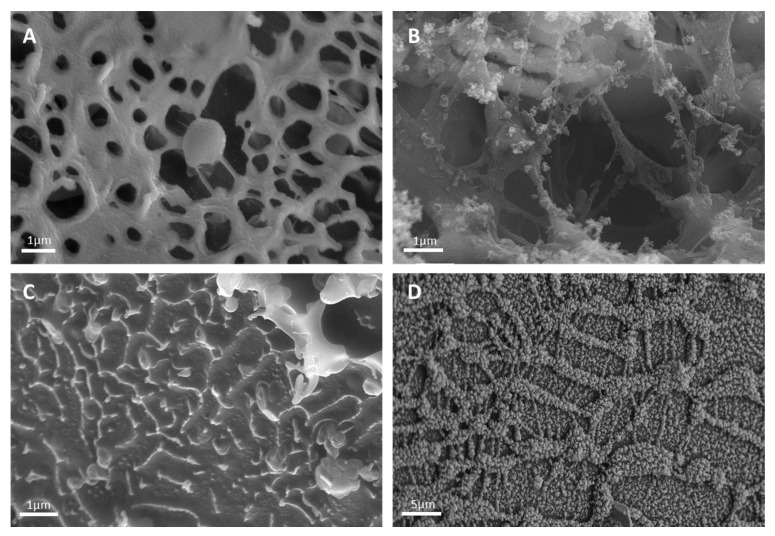
Representative SEM micrographs of freeze fractures that show the microstructure of the mucin network of UWS from a healthy volunteer (**A**), from a patient that received eight radiotherapy fractions (**B**), from a patient that received 28 radiotherapy fractions (**C**) and HA 0.25% (*w*/*w*) (**D**).

Cryo-SEM images showed that UWS formed a coherent network of thick interacting mucin fibers (Figure 4A). The evaluated pore size was between 100 and 2000 nm, with more than 70 percent of the pores showing a diameter between 800 and 1000 nm. In UWS_RT8-16_, the network appeared fragile, as partially broken sections were visible. This also caused the pore size to increase to a maximum of 7000 nm, with the highest volume percentage of the pores showing a diameter between 3200 and 3800 nm. As the fractions of radiotherapy increased, the network became weaker and eventually degraded. UWS_RT25-28_ showed only single non-interacting mucin fibers, i.e., there was no more network available. In contrast, HA 0.25% (*w*/*w*) showed a coherent network. The calculated pore sizes ranged from 80 to 2210 nm. Compared to UWS, fewer pores were visible and the pearl-like fibers appeared thicker. In order to compare our results with previous studies, we used the standard evaluation model, which assumes that the pores are spherical [44,52,62,64]. However, since the pores are not perfectly spherical, the results obtained regarding the pore size and consequently the pore size distribution are only approximations. To obtain more accurate results in the future, other mathematical models should be used, such as the ellipsoid fitting model proposed by Verleysen et al. or the applicability of 3D microscopy as described by Exner should be tested [65,66].

## 4. Discussion

Hypofunction of the salivary gland and xerostomia are long-term consequences of radiotherapy in patients with head and neck cancer, which massively influence the quality of life. As therapeutic approaches, irradiation techniques are modified, stem cell transplantations are discussed, radioprotectors and pharmacological gland stimulators are administered parenterally and saliva substitutes are used [2,67]. However, treatment therapies are still limited due to a variety of disadvantages for the patients. To overcome these limitations, an innovative approach is the development of a topically administered medication that moistens the mouth sufficiently and at the same time transports an active ingredient locally to the salivary glands to protect against irradiation and minimize side effects. As a first approach, however, a sound understanding of the salivary changes caused by radiotherapy is required, as these changes have to be substituted and thus taken into account in the development.

In this study, the saliva of radiation-induced head and neck cancer patients was carefully examined. Independent upon the fractions of radiotherapy, the pH decreased from neutral to acidic, which is in accordance with the literature [22,52,55]. The decreased pH resulted in a slightly lower buffer capacity. Interestingly, commercially available saliva substitutes previously tested by our group, showed a buffer capacity below that of UWS_RT_. This suggests that after administration of these replacement fluids they cannot restore the equilibrium between calcium phosphate of the teeth and the surrounding salivary liquid phase, thus resulting in dentin and enamel demineralization [44,68]. While the osmolality of UWS_RT_ increased significantly in all patients, the electrical conductivity was only slightly increased. Therefore, it can be assumed that the increase in osmolality was not caused by a change in the salivary ionic composition but rather by the loss of water. Aquaporins (AQP), in particular AQP5, which are water channel proteins expressed in epithelial cells in the serous acini of the salivary glands, aid the permeabilization of water [69]. During radiotherapy, it is suggested that there is a loss of AQP5 expression, which limits fluid secretion and hence changes the osmolality. However, the low osmolality of saliva is of paramount importance because it enables the taste buds to perceive various tastes and allows expansion and hydration of mucins [16,70]. Moreover, Simmons et al. showed that hypertonic fluids can cause revocation of residual water from the oral epithelial cells [70]. Hence, an increased osmolality of residual UWS_RT_ might worsen dehydration of the oral epithelium during radiotherapy and increase the risk of taste loss [71]. The osmolality can be further increased by the use of saliva replacement fluids, as these are partly in the isotonic range and show values up to five times higher than healthy saliva [44]. Furthermore, they may contain polymers that do not show sufficient capacity for water absorption. This is of utmost importance to take into consideration as the loss of water is likely to influence the rheology of saliva. In UWS_RT_, the viscosity and the elastic modulus increased resulting in a viscoelastic solid rather than a viscoelastic fluid. However, due to the individuality of saliva, no clear trend could be determined in relation to the radiotherapy fractions. The changes in the viscoelastic moduli and dynamic viscosity between UWS and UWS_RT_ suggest that interactions between the mucins decreased and that the salivary network degraded. This was confirmed by Cryo-SEM. UWS from healthy volunteers showed thick mucin fibers that formed a strong porous network. After two weeks and 8 to 16 fractions of radiotherapy, the fibers became thinner, the network was more fragile and the pores increased in size. After five weeks and 25 radiotherapy fractions, the network was disrupted.

Although the whole protein content was similar between UWS and UWS_RT_, the mucin content was significantly higher in UWS_RT_. This increase was probably the result of reduced water bound within the sample, which resulted in less dilution compared to UWS. Moreover, it is likely that the mucin structure per se changes. Alliende et al. studied the total amount of sulfated oligosaccharides in MUC5B in patients that suffer from Sjögren’s Syndrome and examined that sulfation was decreased [28]. Moreover, Chaudhury et al. found a reduced mucin glycosylation pattern, which showed greater individual variations in MUC5B dependent on the blood group and secretor status [27]. Due to these changes, they concluded that water retention and binding capacities of mucins were reduced, which coincides with our results obtained from the adhesion studies. In general, the adhesive and cohesive properties of material contribute to the wetting and lubrication ability of a fluid such as saliva. While adhesion describes the interaction between two materials, cohesion forces represent the strength of the physical bonds between molecules within a formulation to resist externally applied stress. The adhesion forces of salivary mucins are caused by valence, hydrogen and ionic bonds, with the ionic charges of the glycosylated and sulfated polysaccharide chains being predominant [58]. Due to the limited sample volumes, force curves for UWS_RT_ could not be performed. For UWS, a maximum detachment force of 0.21 ± 0.02 N was obtained. To the authors’ knowledge, there are no comparable human data in the literature. However, Gill et al. determined a maximum force of around 0.31 N for porcine mucin-type III using a lap shear bond test set-up, which is comparable to the tack test [72]. It should be noted here that parameters of the test method such as surface roughness, the surface energy of the substrates and the material of the utilized probe influence adhesion [57,59,73]. This is also valid for atomic force microscopy (AFM), which is currently the most used method in studying force curves [74,75]. However, as only detachment or interactions of single polymer fibers are measured the results are not comparable due to the different size classification.

Biocompatible polymer candidates can be used to replace the protective saliva barrier. Suitable candidates must form a coherent network and exhibit comparable water absorption capacity, wetting behavior and viscoelastic behavior, taking into account the physiological conditions in the mouth. To prove this hypothesis, HA, a polymer that is well-described in the literature, was investigated [30,49,76]. After adjusting the concentration, the viscosity, the elastic moduli and the network formation were close to the values of UWS. Although the pH was slightly higher, which can be considered as an advantage when mixed with diseased saliva, the osmolality was in the isotonic range, thus preventing further revocation of residual water from the oral epithelial cells. The adhesive forces of HA 0.25% (*w*/*w*) were slightly stronger than that of UWS. This is most likely due to the presence of carboxyl and amino groups, which promote strong adhesion by forming hydrogen bonds. Puccio et al. suggest that fractures during stress tests do not occur inside a HA network, but at the polymer–substrate interface [49,77]. This corresponds to the broad curve progression that was observed in comparison to UWS. Therefore, HA can be classified as a well suited topical formulation component to substitute radiation-induced damages in UWS_RT_.

## 5. Conclusions

Summarizing, this study demonstrates that radiotherapy in head and neck cancer patients not only changes the physico-chemical characteristics of saliva but also its network structure and composition. Due to radiation damage to the salivary glands, the electrical conductivity and the total buffer capacity do not change significantly. However, water secretion is reduced, which in turn leads to changes in the pH and osmolality. Although the mucin concentration does not vary significantly, the altered physical properties and the loss of water cause the network to become fragile and eventually degrade, resulting in a loss of the protective barrier. Thus, in order to substitute radiation-induced salivary changes, a saliva replacement fluid must have a neutral to slightly basic pH, low osmolality and a viscoelastic behavior in which G′ dominates, resulting in a tanδ value close to 1. In addition, a stable, coherent network with high water absorption and adhesion properties should be formed to achieve wetting and lubrication in the oral cavity. The investigations showed that HA is a promising candidate that exhibits these properties to recreate the properties of the salivary mucin barrier.

These data will help clinicians build a deeper understanding of the changes in radiation treatment-induced saliva to better evaluate and adapt appropriate saliva substitutes to patients’ needs. In addition, the carefully conducted methods and obtained results will form the basis for technological improvements of new saliva substitutes in pharmaceutical formulation. This will benefit the treatment of dry mouth in the future and support the well-being of patients.

## Figures and Tables

**Table 1:** pH value, osmolality, electrical conductivity and buffer capacity of unstimulated whole saliva (UWS) and UWS_RT_ and hyaluronic acid (HA) 0.25% (*w*/*w*). Differences were evaluated significant at a level of *p* < 0.05, (*), and *p* < 0.001 (***).

Physico-Chemical Characteristics	UWS	UWS_RT_	HA 0.25%
pH	6.76 ± 0.19	6.01 * ± 0.68	7.89 * ± 0.14
Osmolality	0.050 ± 0.013	0.165 *** ± 0.056	0.018 * ± 0.0008
Electrical conductivity	4.73 ± 0.26 ms/cm	5.24 ± 0.54 ms/cm	-
Buffer capacity	5.34 ± 1.7 mmol H^+^/L	4.44 ± 0.56 mmol/H^+^/L	-
